# Protein-based signatures of functional evolution in *Plasmodium falciparum*

**DOI:** 10.1186/1471-2148-11-257

**Published:** 2011-09-14

**Authors:** Kate B Gardner, Ipsita Sinha, Leyla Y Bustamante, Nicholas PJ Day, Nicholas J White, Charles J Woodrow

**Affiliations:** 1 Wellcome Trust Mahidol University-Oxford Tropical Medicine Research Unit (MORU), 420/6 Rajwithi Road, Bangkok, 10400 Thailand; 2 Centre for Infection, St. George's, University of London, Cranmer Terrace, London, UK SW17 0RE; 3 Sanger Malaria Programme, Wellcome Trust Sanger Institute, Hinxton, Cambridge, UK CB10 1SA UK

## Abstract

**Background:**

It has been known for over a decade that *Plasmodium falciparum *proteins are enriched in non-globular domains of unknown function. The potential for these regions of protein sequence to undergo high levels of genetic drift provides a fundamental challenge to attempts to identify the molecular basis of adaptive change in malaria parasites.

**Results:**

Evolutionary comparisons were undertaken using a set of forty *P. falciparum *metabolic enzyme genes, both within the hominid malaria clade (*P. reichenowi*) and across the genus (*P. chabaudi*). All genes contained coding elements highly conserved across the genus, but there were also a large number of regions of weakly or non-aligning coding sequence. These displayed remarkable levels of non-synonymous fixed differences within the hominid malaria clade indicating near complete release from purifying selection (dN/dS ratio at residues non-aligning across genus: 0.64, dN/dS ratio at residues identical across genus: 0.03). Regions of low conservation also possessed high levels of hydrophilicity, a marker of non-globularity. The propensity for such regions to act as potent sources of non-synonymous genetic drift within extant *P. falciparum *isolates was confirmed at chromosomal regions containing genes known to mediate drug resistance in field isolates, where 150 of 153 amino acid variants were located in poorly conserved regions. In contrast, all 22 amino acid variants associated with drug resistance were restricted to highly conserved regions. Additional mutations associated with laboratory-selected drug resistance, such as those in PfATPase4 selected by spiroindolone, were similarly restricted while mutations in another calcium ATPase (PfSERCA, a gene proposed to mediate artemisinin resistance) that reach significant frequencies in field isolates were located exclusively in poorly conserved regions consistent with genetic drift.

**Conclusion:**

Coding sequences of malaria parasites contain prospectively definable domains subject to neutral or nearly neutral evolution on a scale that appears unrivalled in biology. This distinct evolutionary landscape has potential to confound analytical methods developed for other genera. Against this tide of genetic drift, polymorphisms mediating functional change stand out to such an extent that evolutionary context provides a useful signal for identifying the molecular basis of drug resistance in malaria parasites, a finding that is of relevance to both genome-wide and candidate gene studies in this genus.

## Background

Identifying the molecular basis of disease-causing traits is one of the major justifications for the recent expansion in genomic data covering a wide range of taxa. Nowhere is this goal more clearly defined than in the case of malaria, where adaptive evolution in the form of drug resistance continues to undermine efforts to control human disease caused by *P. falciparum *[1] and *P. vivax *[2]. Understanding the molecular basis of resistance phenotypes is of great operational importance as markers can be used to monitor spread and alternative therapeutic strategies can be designed. Establishment of a genetic cross has proven a fruitful starting point for determination of the genotypic basis of drug resistance [3-5], but is difficult, expensive and hence rarely achieved in practice. Genomic epidemiological approaches currently represent a promising route forward allowing detection of signatures of selection associated with drug resistance [6], although this approach relies on identification of linkage disequilibrium which is known to be of variable strength [7]. Candidate gene approaches based on other forms of data analysis may also generate hypotheses suitable for further testing [8,9].

Despite the power of these approaches for the discovery of new drug-resistance genotypes, a decade has passed since the description of the last confirmed resistance gene for a widely used antimalarial [4]. It remains exceptional for mutations hypothesized as being involved in drug-resistance to be validated by transfection and phenotypic testing in *P. falciparum*, steps that allow polymorphisms of true adaptive value to be discriminated from those that are present simply as a result of genetic drift.

A specific phenomenon that may complicate studies in this genus relates to the remarkable degree to which *Plasmodium *proteins are enriched in non-globular domains [10]. Since their first systematic description more than a decade ago, the function of these domains has remained unresolved [11-13] with one possibility being that they represent downstream consequences of events at the DNA level (i.e. broadly neutral in functional terms). If this were to be the case, they would represent potent generators of neutral or near neutral non-synonymous polymorphism in line with central concepts of modern evolutionary biology [14-16] that will inevitably confound both genome-wide and candidate approaches to identifying the molecular basis of resistance. Notably, minimal attempts have been made to determine systematically the degree to which specific areas of coding sequence have become released from negative selective in the long-term, and by inference unlikely to mediate functional change in the short-term. Measurement of selective forces is still generally undertaken at a whole-gene level, an approach that falls down if pressures vary considerably within individual genes, preventing determination of the true baseline for variation which is neutral evolution within and between malaria species.

We reasoned that application of two basic biological concepts could improve significantly the accuracy of approaches designed to identify the genetic basis of drug-resistance. Firstly, drug resistance, by necessity, requires a significant functional change in the parasite, and hence a functional change in one or more proteins. Secondly, functionally important regions of proteins are generally conserved across wide distances of evolution, and can therefore be discriminated in orthologous sequence alignments from sequences under minimal constraint [17].

We undertook a systematic study of evolutionary processes in *Plasmodium *coding sequences, revealing widespread, dynamic variation in selection pressures within individual coding sequences consistent with neutral theories of evolution. The use of underlying conservation score as an independent means of identifying functional variation against the backdrop of genetic drift was validated and applied by examining the molecular basis of drug-resistance, a phenotype that, by necessity, requires a significant functional change in the parasite, and hence frequently a functional change in one or more proteins.

## Results

### Polymorphism and divergence: whole-gene level

In order to study short-term evolutionary processes within the hominid malaria clade a reference set of 40 *P. falciparum *genes (Table 1) was identified, consisting of sequences well covered by genome sequencing of the closely related primate parasite *P. reichenowi *[18]. Single-nucleotide changes in these genes associated with divergence (between *P. falciparum *3D7 strain and *P. reichenowi*) and intra-species polymorphism (within sequenced *P. falciparum *isolates) were quantified. The 40-gene reference set contained 1270 fixed synonymous differences between *P. falciparum *and *P. reichenowi *indicating a synonymous pairwise divergence (dS) of 6.05%, a figure consistent with previous estimates [19]. There were 795 non-synonymous fixed differences (dN = 0.92%) indicating an overall dN/dS ratio of 0.17, consistent with broad negative selection across these genes. This compares to a genome-wide assessment of fixed differences between *P. falciparum *and *P. reichenowi*, dN/dS = 0.21, and indicates that the sequences chosen broadly capture the range of dN/dS ratios encountered in the *P. falciparum *genome [18]. Within sequenced *P. falciparum *isolates there were 83 synonymous and 102 non-synonymous polymorphisms (dN/dS ratio 0.31). Non-synonymous changes hence constitute 55.1% of intraspecies polymorphism but only 38.5% of interspecies divergence, suggesting that approximately one-third of observed amino acid variation within sequenced *P. falciparum *isolates is deleterious and would naturally undergo purifying selection over a longer period.

**Table 1:** Reference set of genes

*P. falciparum *gene	Other identifier	Product	Pf amino acids	*P. chabaudi *gene	Pc amino acids
**ATPases**					
PFA0300c	MAL1P1.52	Vacuolar ATPase-coupled proton transport	383	PCHAS_020560	383
PFC0840w	PfATPase7	Phospholipid transporting ATPase	1864	PCHAS_080610	1773
PFE0195w	PfATPase3	Cation transporting ATPase	2393	PCHAS_110330	1904
PF07_0047	None	Cell-division cycle ATPase	1229	PCHAS_122230	1106
PF08_0113	None	Vacuolar proton transporting ATPase	1053	PCHAS_122440	957
PFI0240c	None	Cu^2+^- transporting ATPase	2563	PCHAS_041740	2128
PFL0590c	PfATPase4	Non-SERCA type Ca^2+^-transporting ATPase	1208	PCHAS_020540	1468
PFL1125w	MAL12P1.225	Phospholipid transporting ATPase	1618	PCHAS_144030	1601
**Transporters**					
PFB0210c	PfHT1	Hexose transporter	504	PCHAS_030470	505
PFB0465c	PF02_0097	Monocarboxylate transporter	457	PCHAS_030940	456
PFE1185w	MAL5P1.237	Metal transporter	684	PCHAS_123900	704
PFF0450c	MAL6P1.94	Zn^2+ ^or Fe^2+ ^transporter	361	PCHAS_010830	338
PFF0170w	MAL6P1.38	Calcium antiporter	441	PCHAS_010300	440
PFF1430c	MAL6P1.133	Amino acid transporter	606	PCHAS_112780	617
PFI1295c	None	Monocarboxylate transporter	529	PCHAS_082700	507
PF11_0210	None	Mg^2+^, Co^2+ ^& Ni^2+ ^channel	529	PCHAS_091640	435
PF11_0338	PfAQP	Aquaglyceroporin	258	PCHAS_093040	258
MAL13P1.23	None	CorA-like Mg^2+ ^transporter	468	PCHAS_140460	491
PF13_0252	PfNT1	Nucleoside transporter	422	PCHAS_136470	414
PF14_0679	None	Inorganic anion antiporter	664	PCHAS_133900	700
**Glycolytic**					
PFF1155w	MAL6P1.189	Hexokinase	493	PCHAS_112240	494
PF14_0341	None	Glucose-6-phosphate isomerase	579	PCHAS_100870	578
PFI0755c	None	6-Phosphofructokinase	1418	PCHAS_081620	1306
PF14_0425	None	Fructose-bisphosphate aldolase	369	PCHAS_131180	369
PF14_0378	None	Triosephosphate isomerase	248	PCHAS_130700	248
PFI1105w	PGK	Phosphoglycerate kinase	416	PCHAS_082320	416
PF11_0208	None	Phosphoglycerate mutase	250	PCHAS_091620	250
PF10_0155	None	Enolase	446	PCHAS_121500	446
PFF1300w	MAL6P1.160	Pyruvate kinase	511	PCHAS_112510	511
PF13_0141	PfLDH	L-lactate dehydrogenase	316	PCHAS_134470	316
**DNA/RNA**					
PFB0730w	PF02_0151	DEAD/DEAH box helicase	1997	PCHAS_031480	1404
PFC0805w	MAL3P6.20	DNA-directed RNA polymerase II	2457	PCHAS_080680	2307
PFE0465c	MAL5P1.95	RNA polymerase I	2914	PCHAS_110870	2570
PFE0715w	MAL5P1.144	Asparagine-tRNA ligase	1128	PCHAS_111360	1084
PFF1095w	MAL6P1.201	Leucyl tRNA synthase	1447	PCHAS_112120	1251
MAL7P1.145	None	Mismatch repair protein pms1	1330	PCHAS_020880	1094
PF13_0251	None	DNA topoisomerase III	710	PCHAS_136460	676
PF14_0234	None	DNA-directed DNA polymerase	1236	PCHAS_101890	989
PF14_0316	None	DNA topoisomerase II	1472	PCHAS_101120	1460
PF14_0695	None	DNA-directed RNA polymerase	861	PCHAS_134050	796

### Conservation across the *Plasmodium *genus

Study of conservation across the *Plasmodium *genus was undertaken using orthologous protein sequences from the rodent parasite *P. chabaudi*, as genome sequencing of *P. chabaudi *was at a more advanced stage at the time of the studies than other available rodent species. *P. falciparum *(3D7) protein sequences were compared with *P. chabaudi*; an average score for all hominid malaria variants would strictly be more accurate, although this would only influence approximately 1% of residues. The mean level of protein conservation per gene ranged widely across the reference set of 40 housekeeping genes (mean +4.88 to -0.47 based on the BLOSUM62 matrix). It was also noted that overall 20.1% of *P. falciparum *residues were non-aligned, compared to 13.3% for *P. chabaudi*, consistent with expansion of non-aligned sequence in the hominid malaria lineage since the last common ancestor of these species.

### Correlation between short and long-term conservation

**Figure 1:**
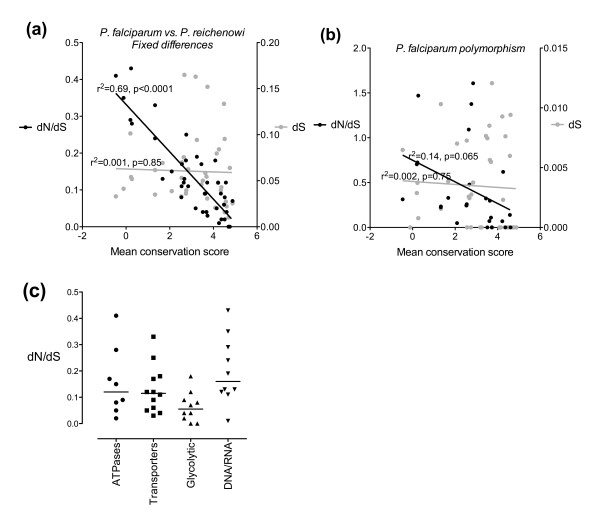
**Hominid malaria clade divergence and conservation across the *Plasmodium *genus**. For the forty individual genes of the reference set, dN/dS and dS are plotted against mean conservation score for each gene. **(a) **Fixed differences between *P. falciparum *and *P. reichenowi*. **(b) **Polymorphisms within *P. falciparum*. **(c) **dN/dS ratios for fixed differences between *P. falciparum *and *P. reichenowi *according to functional group.

At the whole-gene level the dN/dS ratio for *P. falciparum *- *P. reichenowi *divergence strongly correlated with the mean cross-genus conservation score (Figure 1a, r^2 ^= 0.69, p < 0.0001); as expected dS showed no such relationship. For polymorphism within *P. falciparum *(where interpretation of dN/dS is less straightforward [20]), non-significant trends of the same form were seen (Figure 1b). Gene function also appeared to be associated with the degree of purifying selection, with glycolytic enzymes showing lower dN/dS values than the other groups (Figure 1c) consistent with previous work [18]. We compared our dN/dS scores for *P. falciparum/reichenowi *divergence (Nei-Gojobori method with Jukes-Cantor correction) with those previously reported for the same 40 genes using maximum likelihood for calculation of dN/dS [18]. The two sets of results were well correlated (Spearman r = 0.96) indicating that in relative terms dN/dS is very similar for the differing methods; it also confirms that the fixed difference datasets between the two studies are highly comparable. The slope of the line of correlation between our results and those of Jeffares et al. [18] was 0.81 suggesting that the Nei-Gojobori analysis overestimates dN/dS to a small degree compared to PAML.

**Figure 2:**
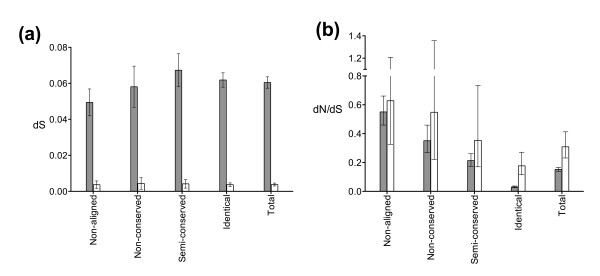
**Mutation rates within the hominid malaria clade stratified by conservation level across the *Plasmodium *genus**. **(a) **Synonymous substitution rates. **(b) **dN/dS ratios. Data are based on 40 reference genes. Fixed differences between *P. falciparum *and *P. reichenowi *are indicated by filled grey bars; polymorphism within *P. falciparum *is indicated by empty bars. Error bars represent 95% confidence intervals. Conservation levels were based on BLOSUM62 score at individual amino acids in *P. falciparum *- *P. chabaudi *alignments; identical (+4 and above), semi-conserved (0 to +3), non-conserved (-4 to -1) and non-aligned.

A stratified analysis was undertaken in which each amino acid residue was assigned to one of four levels of cross-genus evolutionary conservation according to *P. falciparum *- *P. chabaudi *CLUSTALW alignment. Residues were defined as non-aligned, non-conserved (-4 to -1), semi-conserved (0 to +3) and identical (+4 and above) on the basis of the BLOSUM62 matrix. Synonymous substitution rates within the hominid malaria clade were similar across all levels of conservation (Figure 2a) but non-synonymous substitution rates were strongly influenced by long-term evolutionary context (Figure 2b). The dN/dS ratio for divergence was approximately 20-fold higher at non-aligning residues (0.64) compared to residues identical across the genus (0.034; Table 2). Chi-squared testing comparing divergence with polymorphism (McDonald-Kreitman test) within each conservation stratum showed that strong purifying selection was largely confined to residues identical across the genus (Table 2), with minimal evidence for purifying selection in non-aligned sequence regions.

**Table 2:** Divergence and polymorphism in reference genes assessed by McDonald-Kreitman test, stratified by level of cross-genus conservation

Conservation level	dN/dS	Adjusted dN/dS		Synonymous	Non-synonymous	Chi-squared	Neutrality Index
Identical	0.030	0.034	D	839	101	8.3 × 10^-15^	5.6
			P	52	35		
Semi-conserved	0.21	0.24	D	186	174	0.22	1.6
			P	12	18		
Non-conserved	0.35	0.39	D	90	118	0.38	1.5
			P	7	14		
Non-aligned	0.55	0.64	D	155	402	0.74	1.1
			P	12	35		
Total	0.15	0.17	D	1270	795	9.5 × 10^-6^	2.0
			P	83	102		

dN/dS ratios refer to *P. falciparum */*P. reichenowi *divergence. Conservation levels were based on BLOSUM62 score in *P. falciparum *- *P. chabaudi *alignments; identical (+4 and above), semi-conserved (0 to +3), non-conserved (-4 to -1) and non-aligned. Adjusted dN/dS ratios were derived based on the transition: transversion ratio measured at 4-fold degenerate sites (see Table 4). D = divergent mutations, P = polymorphisms. Chi-squared and Neutrality Index are the outputs of the McDonald-Kreitman test; neutrality index indicates the extent to which the levels of variation depart from the expected in the neutral model with values greater than 1 indicating an excess of polymorphism and hence negative selection.

### Relationship between evolutionary conservation, hydrophilicity and informational complexity

Given the lack of a gold-standard informatic approach to defining non-globular regions, the relationship between conservation and non-globularity was explored via one purely structural marker of non-globularity (Kyte-Doolittle hydrophilicity) as well as a purely informational one (low-complexity regions defined by the SEG algorithm). These studies were undertaken in the context of an approach to cross-genus conservation measurement designed for high throughput use, involving a sliding window of 9 amino acid residues.

**Figure 3:**
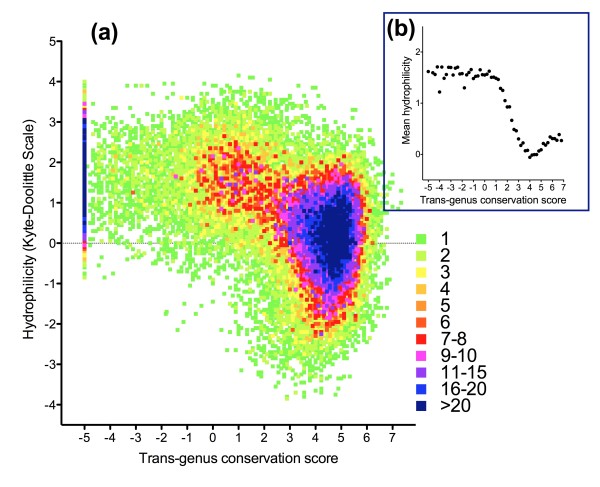
**Density plot of conservation vs. hydrophilicity for amino acid residues of 40 reference genes**. **(a) **Number of occurrences at each co-ordinate (using bins of 0.1 on both × and Y-axes). **(b) **Average hydrophilicity (conservation score bins of 0.2). Conservation levels in *P. falciparum *- *P. chabaudi *alignments were based on BLOSUM62 score using a sliding, overlapping window of 9 residues; hydrophilicity was based on the Kyte-Doolittle index (sliding, overlapping window of 14).

**Figure 4:**
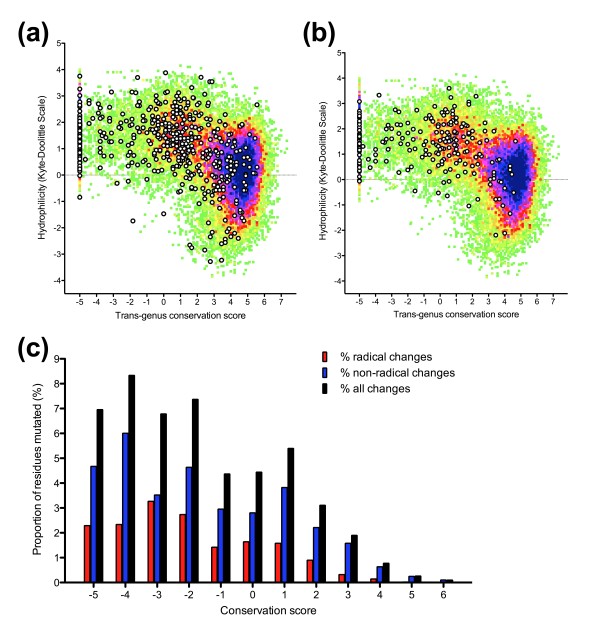
**Conservation-hydrophilicity at sites of amino acid variants in 40 reference genes**. **(a) **Conservative amino acid variants between *P. falciparum *and *P. reichenowi*. **(b) **Radical amino acid variants between *P. falciparum *and *P. reichenowi*. **(c) **Proportion of total sequence, conservative amino acid variants and radical amino acid variants according to conservation level (conservation score bins of 1.0). Conservation and hydrophilicity were derived as for Figure 3.

Decreasing cross-genus conservation was linked to both a shift to unusually high hydrophilicity scores, with a clear inflexion point present between conservation scores of 2 and 3 (Figure 3), and a reduction in strength of negative selection within the hominid malaria clade (Figure 4). A minority (31%) of sequence with conservation score less than 2.5 was responsible for 77% of non-synonymous differences between *P. falciparum *and *P. reichenowi*, including 87% of radical substitutions (Figure 4c). Low-complexity regions defined by the SEG algorithm (default parameters) made up 11.9% of the sequence studied (missing many poorly conserved regions of sequence and including some highly conserved areas), and contained only 19.0% of non-synonymous differences between *P. falciparum *and *P. reichenowi*. Taken together these observations indicate that a measurement of cross-genus evolutionary conservation identifies highly hydrophilic sequences that show relaxed purifying selection in the short-term, features of non-globular domains, and that this method appears superior to an approach based purely on informational complexity.

### Chromosomal regions containing genes responsible for drug resistance in field isolates

**Figure 5:**
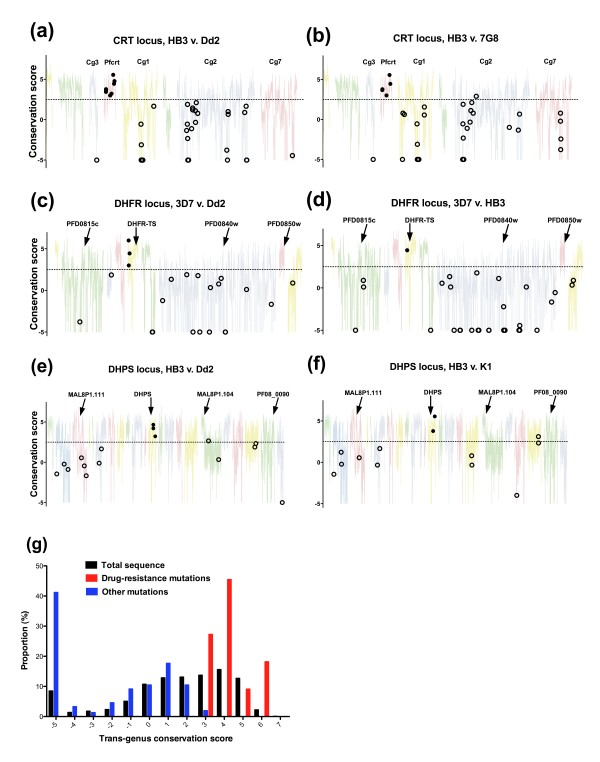
**Relationship between *Plasmodium *genus conservation score and amino acid variation in *P. falciparum *drug-resistance regions**. For each of three drug-resistance regions, two comparisons between resistant and sensitive isolates were made. **(a, b) **CRT resistance locus on chromosome 7. **(c, d) **DHFR resistance locus on chromosome 4. **(e, f) **DHPS locus on chromosome 8. Mutations in known drug-resistance genes are shown as filled circles; other mutations are shown as open circles. A threshold conservation score of 2.5 is indicated by the dotted line. Only coding regions are displayed, with gene designations and colours used for orientation purposes only. **(g) **Proportion of total sequence, amino acid variation in drug-resistance genes and amino acid variation in other genes according to conservation level (based on a sliding window of 9 residues, with conservation scores shown within bins of 1.0).

**Figure 6:**
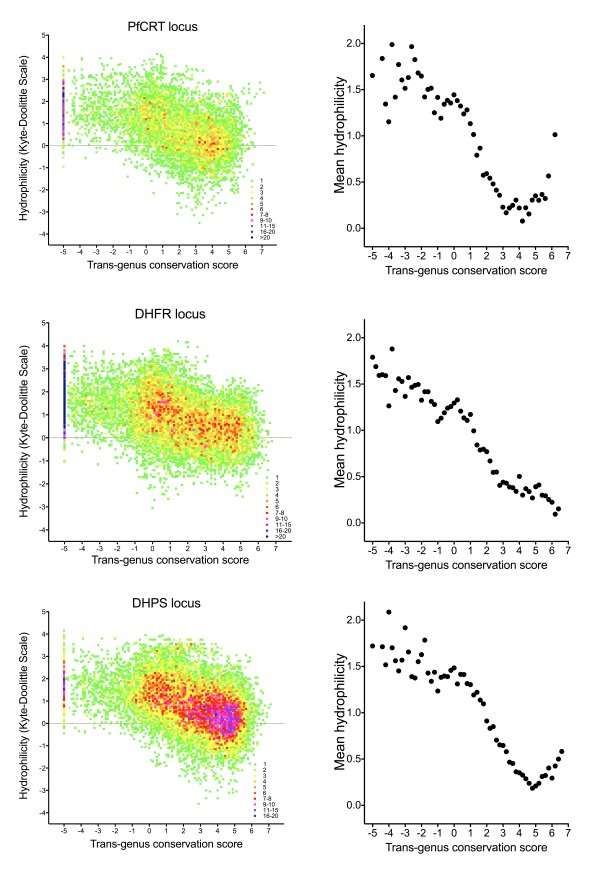
**Density plot of conservation vs. hydrophilicity for 3 drug resistance regions**. **Left**: Number of occurrences at each co-ordinate (using bins of 0.1 on both × and Y-axes). **Right**: Mean hydrophilicity by conservation score (bins of 0.2). Conservation and hydrophilicity are derived as for Figure 3.

In order to test the utility of this approach for detecting functional variation in *P. falciparum*, we examined three chromosomal regions containing *PfCRT*, *PfDHFR *and *PfDHPS*, testing polymorphisms between two pairs of sensitive and resistant parasite isolates in each case. As observed with the reference genes, there were wide ranging levels of long-term conservation (Figure 5), and a similar shift to very high levels of hydrophilicity at conservation scores below 3 in all three cases (Figure 6). Indeed, the proportion of sequence falling below a conservation threshold of 2.5 was even greater than in the reference gene set (56% vs. 31%) indicating that at a genomic level non-globular domains may be responsible for an even higher proportion of non-synonymous polymorphism than in our reference genes. Consistent with this, amino acid variants in genes not involved in drug resistance fell almost exclusively in less conserved regions with 150 of 153 such amino acid variants below the 2.5 conservation threshold (Figure 5g, Chi-squared test for proportion of mutations vs. proportion of total sequence at each conservation level: p = 9 × 10^-23^). In contrast 22 amino acid variants within known drug-resistance genes were located in more conserved regions (Chi-squared test for proportion of mutations vs. proportion of total sequence at each conservation level: p = 3.8 × 10^-7^), with all mutations being located at positions with relatively high conservation scores (> 3.0). Further there was no overlap in conservation scores between amino acid variants in the drug-resistance gene and those within other genes within any single alignment. These results indicate that the position of mutations within a long-term and measurable evolutionary landscape provides a robust signature that can aid identification of functionally important amino acid variants.

### Laboratory-selected drug resistance

**Figure 7:**
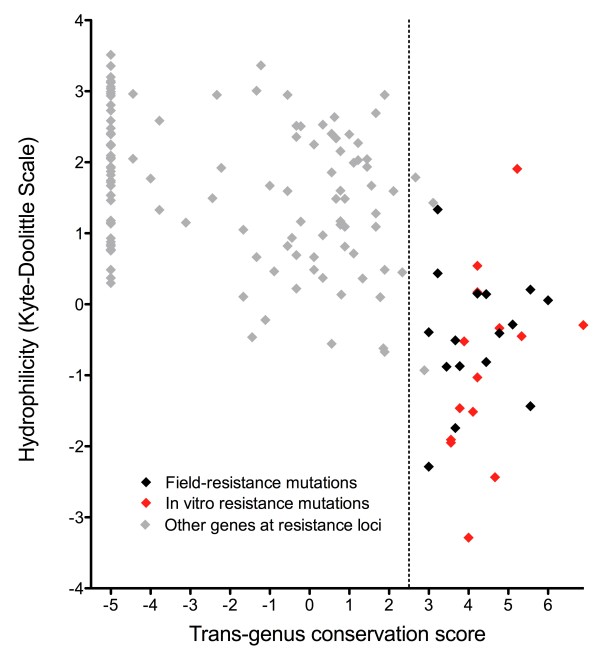
**Conservation-hydrophilicity plot for amino acid variants associated with drug resistance or within drug-resistance regions**. All mutations shown to be involved in drug resistance by transfection (22 nSNPs in CRT, DHFR and DHPS) or drug pressure studies (14nSNPs in cytochrome b, rpl4 and PfATPase4) are shown along with variants in chromosomal regions around CRT, DHFR and DHPS (see Figure 5).

Fourteen amino acid variants associated with selection of atovaquone, azithromycin and spiroindolone resistance in vitro (involving mitochondrial, apicoplast and nuclear genomes respectively [21-23]) conformed to the same pattern (Figure 7). Combining these with field resistance mutations produced a set of 36 non-synonymous polymorphisms associated with drug resistance. Notably, among these mutations the nature of the amino acid variants itself (radical vs. conservative) provided no discriminatory value, indicating that it is primarily the location of the protein change that distinguishes functional adaptation.

### Population data from *P. falciparum *isolates

**Figure 8:**
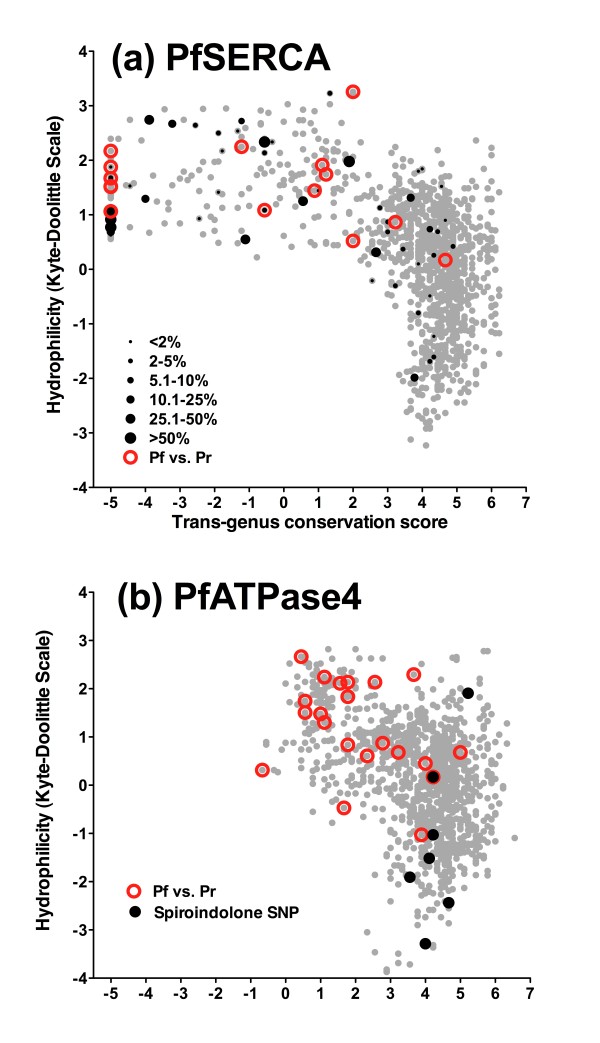
**Conservation-hydrophilicity plots and single-nucleotide changes in the hominid malaria clade in calcium-ATPase genes**. **(a) **PfSERCA (PFA0310c) showing amino acid variants between *P. falciparum *and *P. reichenowi *and field amino acid variants (see Table 3); symbol size indicates maximum allele frequency on a countrywide basis. **(b) **PfATPase4 (PFL0590c), showing amino acid variants between *P. falciparum *and *P. reichenowi *as well as the mutations associated with spiroindolone selection (see text). Conservation and hydrophilicity are derived as for Figure 3.

Evolutionary analysis of long-term conservation is also relevant to population studies on large numbers of field samples since the neutral theory of evolution describes a strong relationship between selective pressure and allele frequency [24]. Sequences of PfSERCA, a postulated target of artemisinin, have been determined for thousands of isolates across the world (See Table 3 for data sources). Consistent with neutral evolution, maximum allele frequencies for amino acid variants in PfSERCA were linked closely to cross-genus conservation level. All mutations reaching a prevalence of more than 25% were located in regions of low conservation (Figure 8a), matching the pattern of fixed differences between *P. falciparum *and *P. reichenowi *SERCAs. The opposite pattern was seen with amino acid variants associated with spiroindolone resistance in PfATPase4, which stand out distinctly against the flow of corresponding amino acid variants between *P. falciparum *and *P. reichenowi *ATPase4 sequences (Figure 8b).

**Table 3:** Maximum field allele frequencies for non-synonymous mutations in PfSERCA (PFA0310c, PfATP6)

Mutation	Frequency (%)	Location	Reference
**R37K**	100	Venezuela	[54]
**K67R**	3	Ghana	[54]
**K88E**	1	Tanzania	[54]
**I89T**	38	Philippines	[54]
**S176N**	3	Malawi	[54]
**V229I**	< 1	Zanzibar	[55]
**I230T**	3	Iran	[54]
**H243Y**	5	Malawi	[54]
**A338V**	1	Tanzania	[54]
**L402V**	28	Brazil	[56]
**D405N**	1	Tanzania	[54]
**E431K**	58	Ethiopia	[57]
**E432K**	36	Cameroon	[58]
**D436N**	3	Tanzania	[54]
**A438D**	18	Vanuatu	[54]
**N462D**	10	Venezuela	[54]
**N463S**	3	Vietnam	[59]
**464 del**	2	Cambodia	[60]
**N465S**	16	Thailand	[1]
**S466N**	35	Peru	[61]
**S529I**	3	Tanzania	[54]
**K561N**	1	Niger	[62]
**N569K**	36	Zanzibar	[55]
**G585D**	< 1	Zanzibar	[55]
**T602I**	< 1	Zanzibar	[55]
**L610I**	1	Tanzania	[54]
**K611N**	< 1	Zanzibar	[55]
**N612D**	< 1	Zanzibar	[55]
**A621D**	< 1	Zanzibar	[55]
**A623E**	4	Ethiopia	[57]
**A630S**	58	Brazil	[56]
**G632E**	3	Malawi	[54]
**T635K**	16	Madagascar	[54]
**E637G**	< 1	Zanzibar	[55]
**G639D**	90	Venezuela	[54]
**S641G**	< 1	Zanzibar	[55]
**E643Q**	1	Zanzibar	[55]
**N644I**	1	Zanzibar	[55]
**K649E**	< 1	Zanzibar	[55]
**L650W**	2	Bangladesh	[54]
**T657I**	3	Malawi	[54]
**N683K**	9	Bangladesh	[54]
**I723V**	3	Tanzania	[54]
**H747Y**	11	Madagascar	[54]
**S769N**	4	Ethiopia	[57]
**K771A**	4	Angola	[63]
**K776N**	1	Angola	[63]
**K783E**	1	Tanzania	[54]
**G805E**	3	Malawi	[54]
**R809G**	< 1	Zanzibar	[55]
**844 del**	77	Peru	[61]
**D845N**	3	Tanzania	[54]
**E847K**	2	Thailand	[1]
**T857S**	1	Tanzania	[54]
**A1115G**	4	Papua New Guinea	[54]
**V1169I**	24	Brazil	[54]
**S1187T**	3	Ghana	[54]
**D1228E**	3	Ghana	[54]

3D7 sequence was used as reference. Only studies where alleles were determined for at least ten samples were included. Samples with mixed infections were included in each numerator.

## Discussion

### Non-adaptive evolution in *Plasmodium*

We show that genes that maintain synteny and clearly defined orthologous relationships across the genus also contain poorly conserved domains that occupy approximately half of the total coding sequence when analysed at the chromosomal level. In other words, in *Plasmodium *species, negative (purifying) selective force is unusually chimeric in nature, being alternately strong and weak within the same gene. *Plasmodium chabaudi *has approximately two-thirds as much non-aligned sequence as *P. falciparum*, while other work on a genome-wide scale indicates that *P. vivax *has approximately 40% [25]. This indicates that the phenomenon is genus-wide, being particularly marked in the hominid malaria clade. The reason why internal proteins mediating core biological functions possess such widespread areas of non-globular domain experiencing minimal purifying selection remains obscure but our data are certainly consistent with these sequences representing a downstream consequence of genetic processes [13,26] such as replication slippage and unequal crossing over [27]. The dN/dS ratio of 0.64 in non-aligned regions suggests that these sequences are under very weak negative selective pressure, with purifying selection perhaps acting only to maintain non-globular structure.

### Applications

Improved understanding of these issues has major implications for the study of all *Plasmodium *biology that relates to evolution. The findings are most immediately relevant to the goal of understanding molecular mechanisms of drug resistance, since this represents a classical example of evolution in action. As predicted, we were able to show that mutations selected by drug pressure in the field or laboratory are located in conserved protein sequences that have remained largely unchanged for millions of years (being conserved across the *Plasmodium *genus) by virtue of their functional importance, as already noted by other authors [22,28-30]. What has not been considered is that the gradient in purifying selective force across the genus is strong enough to allow drug-resistance mutations to be discriminated accurately from those that are likely to non-adaptive in nature. Prediction of the functional consequences of mutations based on long-term evolutionary conservation has been described in the context of human genetic studies [31-33] but there is even greater scope for this approach to assist studies in *P. falciparum*, where the contribution of non-adaptive change appears to be log-orders of magnitude higher.

The potential for non-adaptive evolution to confound studies of genotype-phenotype relationships is clear in several areas. Candidate gene surveys based on sequencing of field isolates, where few or no phenotypic data are available, provide an obvious example; the majority of polymorphisms found in PfSERCA are seen to be located in poorly conserved regions, including all those reaching frequencies of more than 25% in field isolates. This finding indicates that polymorphism in this gene is dominated by non-adaptive evolution, and the temptation to invoke positive selection [34] should be avoided. Use of dN/dS to infer positive selection in whole genome analyses of *P. vivax *[35] may also be prone to the same issue; although measures of dN/dS across a gene tend to be conservative due to purifying selection, it is still likely on statistical grounds that across a large number of genes, a number of high dN/dS ratios (greater than one) will be generated simply by the action of non-adaptive evolution.

Understanding of the degree to which non-adaptive evolution occurs is also relevant to the identification of drug-resistance genes in a chromosomal context. In our analyses of regions known to contain drug-resistance genes, application of evolutionary conservation added considerable specificity with more than 95% of mutations outside drug-resistance genes falling into poorly conserved sequence. Nevertheless the accuracy of the evolutionary approach as it stands is unlikely to be sufficient for its use in isolation, but rather as a method for pinpointing polymorphisms within regions identified by other means, particularly genome-wide association studies using population genetic tests [6,36]. Genetic cross experiments are also a robust starting point; for example, the complex polymorphisms in the *cg1 *and *cg2 *genes seen at the chloroquine resistance locus identified by the cross of HB3 and DD2 [37] would be defined as being of low priority for further study by the approach we describe. Further work is in progress to examine systematically the evolutionary properties of other regions already identified as being of phenotypic importance by experimental means [38], with the aim of prioritizing polymorphisms for more detailed studies.

### Wider relevance

Awareness of the chimeric selection landscape of *Plasmodium *coding sequences is also relevant to the study of the history of *Plasmodium *species. A higher dN/dS at the whole gene level for hominid parasites *P. falciparum *and *P. reichenowi *compared to rodent malaria parasites has been suggested to provide evidence for small population size during the *P. falciparum*-*reichenowi *divergence [39]. However, based on the set of forty reference genes we studied, hominid malaria proteins contain significantly more non-aligned sequence than do rodents, likely leading to higher dN/dS ratios at the whole gene level. In fact in sequence regions conserved across the *Plasmodium *genus the average dN/dS ratio for the *P. falciparum*-*P. reichenowi *divergence was 0.034, indicating strong purifying selection and implying a historically large population size following the lateral transfer of *P. falciparum *to humans from bonobos [40] or gorillas [41].

## Conclusion

The coding sequences of *Plasmodium *species exemplify the theories of neutral evolution [14,42] and the ensuing nearly neutral theory [15], operating on a grand scale that to our knowledge is unrivalled in genome biology. Failing to take into account the effect of this in any work that relates to evolution of coding sequence represents a missed opportunity to distinguish adaptive from non-adaptive polymorphism. For example neither genome-wide or candidate gene association studies into antimalarial resistance currently take into account the fact that most polymorphism is non-adaptive in nature. The approach we describe takes advantage of protein sequence data, but does not require any knowledge of protein function, a major advantage since the elucidation of function for the majority of *Plasmodium *genes remains a distant goal. Application of this method on a genome-wide scale, where it can be integrated into DNA-based methods as well as candidate gene approaches, offers a powerful approach for future studies in the field of antimalarial resistance, as well as other areas of research that are linked to evolutionary biology in this important genus.

## Methods

### Comparisons between *P. falciparum *and *P. chabaudi *orthologs

The rodent malaria species *Plasmodium chabaudi *was used as comparator species given its status in terms of genome completion. Protein and DNA sequences (Plasmodb version: 2009.03.24) were obtained from Plasmodb.org. Forty orthologous pairs of sequences were chosen, fulfilling the following criteria; no known or hypothesised role in drug resistance or host interaction, syntenic relationship between *P. falciparum *and *P. chabaudi *and > 75% coverage in the *P. reichenowi *ortholog [18] (used for measurement of divergence within hominid malaria parasites). Since sexual-stage genes are released from purifying selection in asexual culture (experienced by several of the isolates under study) [43] genes with no evidence of asexual expression in transcriptomic surveys [44,45] were also excluded. In order to reflect the types of genes which are implicated in drug resistance, as well as to obtain a range of conservation levels across this reference set, the reference genes consisted of ATPases (8), secondarily active transporters (12), glycolytic enzymes (10) and enzymes involved in DNA and RNA processing (10)(Table 1). The Plasmodb gene model for PfL0590c (PfATPase4) was found to be inconsistent with published data based on cDNA; the latter were used as coding sequence [23].

CLUSTALW alignments of orthologous protein sequences from *P. falciparum *and *P. chabaudi *were performed using the default settings of BioEdit. BLOSUM62 scores (reflecting conservation) were then calculated for each *P. falciparum *residue. Regions that could not be aligned between *P. falciparum *and *P. chabaudi *orthologs were defined as the gaps in BLASTP alignments of *P. falciparum *and *P. chabaudi *orthologs (BLOSUM62 matrix, gap penalties: existence 9, extension 2) A manual check of the protein alignment in BioEdit was also performed and on rare occasions where short alignments had been excluded by the BLASTP search these were retained. For residues where there was no aligned *P. chabaudi *residue, a conservation score of -5 was applied. Relatively small regions with no *P. reichenowi *coverage were also removed from analysis to ensure comparable denominators for inter- and intra-species comparison.

### Annotation of polymorphisms and fixed differences

We analysed single-nucleotide polymorphisms (SNPs) derived from Plasmodb, based on available sequence for various *P. falciparum *strains from around the world generated by the Broad Institute [46], Wellcome Trust Sanger Institute [18] and NIH [47]. Fixed differences between *P. falciparum *3D7 strain and *P. reichenowi *(Oscar strain) were also obtained from Plasmodb [18]. Radical amino acid substitutions were defined as those with BLOSUM62 matrix score < 0.

The challenge of identifying single nucleotide changes within a sequence that is undergoing frequent insertion-deletion polymorphism has been described [48]. In addition, we noted that although complex polymorphisms were said to be excluded in publications, the Plasmodb lists of SNPs within *P. falciparum *and fixed differences between *P. falciparum *3D7 strain and *P. reichenowi *sometimes contained repetitive mutations within tandem repeats (confirmed by Pustell protein matrix, MacVector) that were clearly part of complex indel polymorphisms and hence not genuine SNPs. These regions contributed 27.5% of all SNPs among *P. falciparum *isolates and 9.4% of the interspecies divergence, and were excluded from both polymorphism and divergence analyses.

### Calculation of positions and transitions

Calculation of synonymous and non-synonymous positions was undertaken for each *P. falciparum *ortholog using a standard substitution matrix (assuming equal mutation rates) with Jukes-Cantor correction [49]. Confidence intervals for dS were determined assuming a continuous distribution. Confidence intervals for dN/dS were determined using the delta method. Transitional bias was determined by studying synonymous fixed differences between *P. falciparum *and *P. reichenowi *orthologs occurring at amino acids encoded by four codons. Consistent with previous measurements on a chromosomal scale [50], synonymous sites made up 20.1% of all sites within the reference genes (See Table 4) with 8.9% of sites being 4-fold degenerate sites (nucleotide positions at which all mutations are synonymous). For synonymous differences at 4-fold degenerate sites, which we assume are selectively neutral, transitions made up 41.4% of changes and transversions 58.6%, consistent with a moderate transitional bias that would produce falsely low dN/dS ratios [51] (since transitional mutations are associated with degeneracy at many 2-fold degenerate sites). Taking this factor into account led to upwards revision of dN/dS ratios for divergence of between 12 and 16% according to the level of conservation; the effect was greatest for non-aligned sequence where the adjusted dN/dS ratio rose to 0.64 (Table 1).

**Table 4:** Synonymous sites in reference set of genes

Conservation level	*All*	*4-fold*	*Transitional sites*	*Transversional sites*	*Adjusted S*
Identical	14127 (20.8)	6384 (9.4)	6389 (9.4)	1354 (2.0)	15537
Semi-conserved	2889 (19.1)	1249 (8.3)	1319 (8.7)	321 (2.1)	3176
Non-conserved	1609 (21.5)	811 (10.9)	672 (9.0)	126 (1.7)	1759
Non-aligned	3240 (17.7)	1187 (6.5)	1819 (10.0)	234 (1.3)	3658

Total	21866 (20.1)	9631 (8.9)	10200 (9.4)	2036 (1.9)	24131

Transitional sites refer to 2-fold synonymous codons by virtue of transitional substitution; transversional sites to 2-fold synonymous codons by virtue of transversional substitution. Numbers in parentheses indicate proportion (%) of total positions.

### Studies of hydrophilicity and complexity

Hydrophilicity scores were measured by the Kyte-Doolittle index (window = 14). Low-complexity regions were defined using the SEG algorithm at its default parameters [52].

### Drug-resistance chromosomal regions

*P. chabaudi *orthologs were again used to generate the conservation score. In the case of one gene (MAL8P1.111) there was no rodent malaria parasite ortholog as previously reported [53] and in consequence the syntenic *P. vivax *ortholog was used for comparison. For the single apicoplast gene rpl4 the partial *P. chabaudi *sequence PC103611.00.0 was available for generation of the cross-genus conservation score at sites of mutation. For all studies at drug-resistance regions a neighbourhood conservation score was used (averaging the individual conservation scores across a sliding, overlapping window of 9 residues). This allows for the possibility that drug-resistance mutations may occur at residues that have previously undergone conservative change within a wider area of conservation, thereby reducing stochastic loss of sensitivity. This also obviated the need for a specific step to identify non-aligned regions at genomic regions. Non-synonymous SNPs (nSNPs) between sensitive and resistant parasites were studied at each locus, spanning the drug-resistance gene in each case and extending outwards symmetrically until 10 nSNPs outside the drug-resistance gene itself had been documented in at least one pairwise comparison. All residues known to be intrinsic to resistance haplotypes, whether or not each individual residue has been shown to cause drug-resistance independently, were included. Chi-squared testing was undertaken testing whether the distribution of amino acid variants in terms of conservation level was the same as the distribution of total sequence, using 13 conservation levels (bins of 1), and hence 12 degrees of freedom. The test was performed first for drug-resistance genes and mutations, and then for the other genes and the mutations within them.

## Competing interests

The authors declare that they have no competing interests.

## Authors' contributions

CW, KG, LB, ND and NW conceived the study design. KG, IS and CW performed data acquisition, coding and analysis. KG and CW produced the first draft of the paper and all authors amended the manuscript and approved the final version.

## Authors' information

CW, ND and NW are clinicians based at the Mahidol-Oxford Tropical Medicine Research Unit and are engaged in tackling the problem of multi-drug resistant malaria in Southeast Asia.
